# Teachers’ Practices in Developing Entrepreneurial Competence for Innovative Quality Education

**DOI:** 10.3390/ejihpe15060104

**Published:** 2025-06-07

**Authors:** Andrea Gracia-Zomeño, Eduardo García-Toledano, Ramón García-Perales, Ascensión Palomares-Ruiz

**Affiliations:** Department of Pedagogy, University of Castilla-La Mancha, 13071 Ciudad Real, Spain; eduardo.gtoledano@uclm.es (E.G.-T.); ramon.garciaperales@uclm.es (R.G.-P.); ascension.palomares@uclm.es (A.P.-R.)

**Keywords:** entrepreneurial competence, pedagogical innovation, teachers’ practices, teacher training, quality education, active methodologies, key competencies

## Abstract

Entrepreneurial Competence (EC) is increasingly recognized as essential to foster innovation and enhance educational quality. This study explores how education professionals perceive and develop the EC within educational centres, using a qualitative approach through structured interviews with education professionals. The findings highlight the need for a more explicit and systematic development of the EC, emphasizing continuous teacher training in entrepreneurial approaches. While digital and paper-based resources support the EC, educators stress the importance of active and practical methodologies such as Project-Based Learning and Learning by Doing. Limited collaboration among educators is identified as a barrier to effectively foster EC. Moreover, the lack of strategic planning within educational institutions remains an obstacle. This study underscores the importance of introducing structured and innovative pedagogical approaches to ensure that students cultivate the skills necessary to navigate an evolving and uncertain future.

## 1. Introduction

Entrepreneurship education has been consolidated as an essential component in current educational systems, especially in the European context. In the document “Education for Citizenship-Guidelines” of the Directorate-General for Education, Entrepreneurship Education is defined as follows:

Entrepreneurship Education (EE) aims to promote the acquisition of knowledge, skills and attitudes that encourage and ensure the development of ideas, initiatives and projects, in order to create, innovate or make changes in an individual’s life in response to the challenges set by society. EE is a transversal contribution to different subjects and non-subject areas, accomplished through activities or projects, which are developed in a participatory way by students willing to make a change in their capacity as citizens.

(European Commission, 2016, p. 182)

In this context, it is important to distinguish between “Entrepreneurship Education”, which refers to the broader educational approach that fosters entrepreneurial thinking and behaviour (European Commission, 2016), and “Entrepreneurial Competence”, which is a specific construct composed of interrelated knowledge, skills, and attitudes, defined as the ability to turn ideas into actions that generate value for others (Bacigalupo et al., 2016; Council of the European Union, 2018).

The EC is formally recognized as one of the eight Key Competences for Lifelong Learning, updated in Council Recommendation of 22 May 2018, which guides educational strategies in all EU countries. These competences are: (1) Literacy competence; (2) Multilingual competence; (3) Mathematical competence and competence in science, technology, engineering; (4) Digital competence; (5) Personal, social and learning to learn competence; (6) Citizenship competence; (7) Entrepreneurial competence; and (8) Cultural awareness and expression competence (Council of the European Union, 2018).

**Figure 1:**
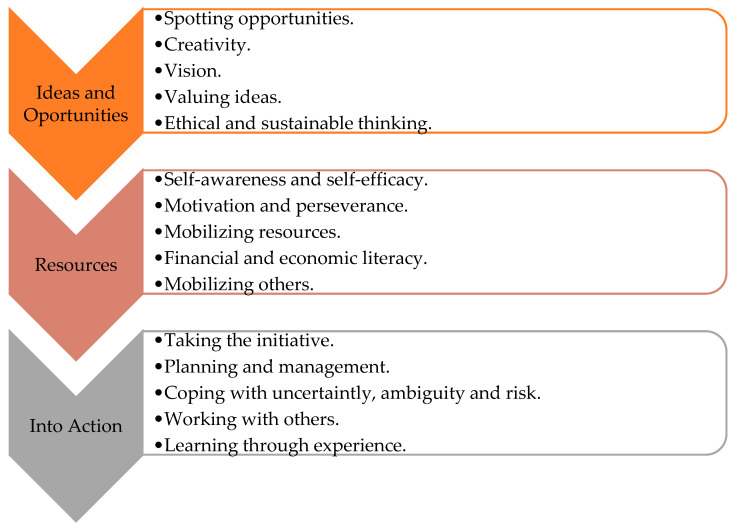
EntreComp: Areas and building blocks. Adapted from Bacigalupo et al. (2016, p. 11).

To define and develop the EC in educational contexts, the European Commission introduced the EntreComp Framework, structured into three interrelated areas—”Ideas and Opportunities”, “Resources” and “Into Action”— composed of 15 “building blocks”, as illustrated in Figure 1 (Bacigalupo et al., 2016).

Since the first efforts to establish a regulatory framework, such as the 2001 Council Conclusions of the European Union, the EU has produced a set of guidelines to support the promotion of entrepreneurship in education and training. These guidelines seek to position entrepreneurship as a key competence, encouraging EU citizens to become entrepreneurs, to generate innovative solutions to social problems and to design products with added socio-economic value (Council of the European Union, 2001).

Entrepreneurship receives significant support through various initiatives of the Erasmus+ programme, aimed at students, trainees or participants in strategic partnership projects outside their home country (European Commission, 2025a). In addition, several tools have been created to facilitate the strengthening of an entrepreneurial mindset, of which HEInnovate is one of the most prominent. This tool targets higher education institutions, promoting innovation and entrepreneurship within universities (European Commission, 2025b).

EU adult education programmes also incorporate tools to support lifelong learning initiatives and enhance education in different sectors, such as the social economy and innovation. Specifically, EU policy initiatives, such as the “European Skills Agenda” and the “European Education Area”, underline the importance of investing in adult skills and lifelong learning, with an emphasis on entrepreneurship and innovation, as key components of adult education (European Commission, 2021a). The integration of these tools into adult learning environments has proven beneficial, as adult learners tend to have a greater connection to real-world contexts, making them more receptive to experiential learning techniques (Council of the European Union, 2020; Merriam & Bierema, 2013).

Beyond institutional support, research has increasingly focused on entrepreneurial intention, which is defined as a state of mind that prepares individuals to embark on new entrepreneurial activities (Osorio & Londoño, 2015). This intention is influenced by cognitive and social elements, with entrepreneurial self-efficacy being one of the most studied factors (Alvites-Huamani et al., 2020; Duran-Aponte & Arias-Gomez, 2015). Optimism and social responsibility have a significant positive impact on entrepreneurial self-efficacy and contribute to determining individuals’ entrepreneurial intention (Cardella et al., 2024).

In recent years, research has considerably broadened our understanding of entrepreneurial intention among students in higher education institutions (HEIs). Some studies have examined the impact of psychological need satisfaction, proactivity and optimism on students’ entrepreneurial intentions (Emami et al., 2021; Na et al., 2022). Other research has investigated the role of Entrepreneurship Education, highlighting how the quality of education and active methods (also known as innovative methods) contribute to the growth of entrepreneurial intentions (Cardenas-Gutierrez et al., 2023; Martinez-Gregorio & Badenes-Ribera, 2021). In addition, the influence of university support in fostering entrepreneurial intentions has been analysed, emphasizing the importance of institutional environments that provide access to resources and facilitate connections with the business sector (Abdelfattah et al., 2022; Pham et al., 2023). Environmental factors, such as perceived social support, have also been found to be crucial in shaping entrepreneurial intentions (Diaz-Casero et al., 2012; Ngek, 2020). Building on the concept of entrepreneurial intention, the cultivation of transversal competencies has emerged as a key factor in strengthening entrepreneurial education, particularly within the framework of the social economy (Gallegos et al., 2024). Increasing emphasis has been placed on training skills, such as creativity, resilience, and decision making (Araya-Pizarro & Aviles-Pizarro, 2020). However, it is essential to clarify that while these elements are sometimes referred to as “skills”, within the competence-based approach, they are components of a broader competence.

Entrepreneurial Competence refers to the capacity to act upon opportunities and ideas, and to transform them into values for others. It is founded upon creativity, critical thinking and problem solving, taking initiative and perseverance and the ability to work collaboratively in order to plan and manage projects that are of cultural, social or financial value.

(Council of the European Union, 2018, p. 11)

Recent documents, such as the Social Economy Action Plan “Building an economy that works for people [sic]: an action plan for the social economy”, highlight the need to strengthen the capacities of social entrepreneurs through education and targeted training in key competencies. This plan advocates the integration of the social economy into education systems, promoting pedagogical strategies that help students grow technical and cross-cutting skills that are essential to address social and economic challenges (European Commission, 2021b). In this context, it is important to distinguish between “training” and “pedagogical strategies”. “Training” refers to structured professional development programmes designed to improve teachers’ knowledge and instructional skills (Fernandes et al., 2023). In contrast, “pedagogical strategies” refer to the broader teaching methods used in the classroom to promote the development of the EC. These strategies include problem-based learning, experiential learning, and reflective practices aligned with the goals of Entrepreneurship Education (Montes-Martinez & Ramirez-Montoya, 2023; Venesaar et al., 2022).

Along these lines, the European Business Promotion Awards highlight initiatives that foster entrepreneurship, particularly those that support the development of skills such as social self-efficacy, the ability to interact effectively with the environment and take advantage of opportunities (European Commission, 2024).

To effectively apply the EC, various methodologies have proven useful, such as case studies, peer learning, problem-based learning, and business simulations (Asuncion, 2019; Felices & Lopez, 2022). These pedagogical approaches are consistent with the Council Conclusions on Entrepreneurship in Education and Training, which emphasize the need to cultivate creativity, autonomy, decision making, and critical thinking within educational practices (Council of the European Union, 2015).

In line with these pedagogical approaches, higher education institutions play a crucial role in the promotion of entrepreneurship by fostering an educational environment that integrates innovation and experiential learning (Martina & Göksen, 2020; Zambrano et al., 2023). In the face of rapid social and economic transformations, universities must adopt pedagogical innovations that enhance students’ EC, while ensuring the relevance and adaptability of educational programmes (Sanz, 2022; Troncoso et al., 2022). The EC aids future professionals in higher education by providing the necessary tools to drive social and economic progress (Gallegos et al., 2024). Educational institutions are increasingly exploring active methodologies to enable students to engage with real-world challenges, fostering autonomy, resilience, and creativity (Asuncion, 2019; Granados et al., 2020). In addition, digital technologies and collaborative learning strategies have been proven effective in strengthening the EC (Haleem et al., 2022; Kurtishi-Kastrati & Thanasi-Boçe, 2023). However, while these approaches offer promising results, their impact is often hampered by the absence of structured and strategic development of the EC in educational centres (Tennakoon et al., 2020).

Given these challenges, this article aims to explore how education professionals perceive and develop the Entrepreneurial Competence in educational centres.

## 2. Materials and Methods

This study is framed within observational research, with a cross-sectional design and qualitative approach. As an observational study, it is based on the collection and analysis of data without manipulation of variables (Gomez-Nuñez et al., 2020). “No interventions are carried out by the investigator” (Mann, 2012, p. 39). In addition, the cross-sectional design implies that data are collected at a single point in time (Lindstrom, 2010). A qualitative approach was adopted to explore how education professionals perceived and developed EC in educational centres.

Data was collected through a structured interview consisting of 8 open-ended questions (labelled Q1 to Q8), divided into 2 sections, and conducted via Google Forms. Participants completed the interview individually in a private setting of their choice, such as their home or workplace, using their personal devices. Participation was completely anonymous and voluntary. The approximate duration of the interview was between 15 and 20 min. No financial or material benefits were offered to participants; however, they were informed that their contribution would support scientific research and knowledge development in the field of education. The estimated invited-to-participant ratio was approximately 3:1.

To ensure the validity of the instrument, a validation process was carried out with 16 academic experts coming from different educational levels and areas of specialization. They assessed the relevance, clarity and pertinence of the questions, resulting in a high Content Validity Ratio (CVR) (Roebianto et al., 2023). Specifically, seventy-five percent of the questions obtained a perfect CVR of 1, while the remaining questions had values close to 1 (0.94), demonstrating the high quality of the instrument.

The sample consisted of 623 anonymous volunteer education professionals from various countries, selected through non-probability convenience sampling. The interview was distributed through institutional channels, academic mailing lists, and professional networks to reach a diverse pool of subjects. Of the participants, 312 were men and 311 were women. The sample included education professionals from various educational levels, ranging from primary to higher education, in order to gather a broad spectrum of perspectives from different educational contexts. Specifically, the sample consisted of Directors (3%), Researchers (10%), Primary School Teachers (21%), Counsellors (1%), Secondary School Teachers (5%), University Teachers (47%) and Secretaries (2%). In addition, 11% of participants were classified in the “Other” category, which includes professionals such as private teachers, teachers in educational academies and other educators who do not belong to the institutional categories listed above.

An inductive approach was followed with the help of the ATLAS.ti tool. This approach was selected in order to allow categories and codes to emerge directly from the participants’ responses, rather than imposing a pre-existing framework (Azungah, 2018).

To ensure the rigor of the research, the study followed the recommendations of the COREQ (Consolidated Criteria for Reporting Qualitative Research) checklist, which promotes transparency in qualitative reporting (Tong et al., 2007). Although there was no direct interaction between researchers and participants, anonymity and confidentiality were fully guaranteed throughout the process. Data saturation was reached when no new categories or relevant information emerged during the coding process, which occurred after analysing approximately 85% of the responses. Triangulation was carried out through the collaboration of four researchers, who coded the data independently and then jointly refined the category system, ensuring consistency and analytical validity.

In addition, Lincoln and Guba’s criteria were applied to reinforce the trustworthiness of the study. Credibility was supported by prior expert validation of the instrument and clear documentation; transferability by detailed description of the context and participants; dependability by the use of ATLAS.ti and a systematic protocol; and confirmability by detailed recording of all decisions and steps taken during the research, ensuring transparency and minimizing personal bias (Enworo, 2023).

Based on the results of the analysis and categorization of the responses, a total of 327 quotes were identified, distributed across 26 emerging codes, and grouped into five main categories. The research was divided into two sections based on these categories.

The first section of the study focused on the first three categories: (1) Knowledge and Perception of the EC (Q1); (2) Teaching of the EC (Q2 and Q3); and (3) Activities and Development of the EC (Q4). In this first section, 257 quotes grouped in 19 codes were identified. The second section of the study focused on the last two categories: (4) Teacher Training and Collaboration in Entrepreneurship (Q5 and Q6); and (5) Teacher Performance in relation to Entrepreneurship and Leadership (Q7 and Q8). In this second section, 70 quotes grouped into 7 codes were identified.

In this article, we focus exclusively on the first section of the study. Table 1 presents the distribution of questions, categories, codes and quotes corresponding to this section.

## 3. Results

### 3.1. First Category of the Interview: Knowledge and Perception of the Entrepreneurial Competence

The first category of the interview, consisting of six codes and 85 quotes (see Table 2), focused on exploring the participants’ knowledge and perception of the EC.

Based on the results obtained, it can be indicated that participants express their interest and curiosity (25 quotes) in obtaining more information about the EC.

*I am not completely familiar with teaching entrepreneurial competence. However, I have a strong curiosity about it. I am intrigued to discover how the development of this competence can influence my entrepreneurial skills and the skills of my pupils.*

Primary School Teacher

*Entrepreneurial Competence is a very present concept nowadays, but I don’t have much information about it. I would like to explore it more and to carry out initiatives on Entrepreneurship Education.*

Secondary School Teacher

*Although I have not explored this competence very much during my work experience (which is not very extensive), hearing about it during my university education aroused my interest in discovering how to apply the skills it integrates in the real world.*

Research Staff

*I have heard about Entrepreneurial Competence many times. In fact, I often wonder what opportunities this competence might afford me in the future and even what opportunities it could offer in the classroom.*

University Teacher

This engagement and interest can be linked to the acquisition of theorical knowledge (19 quotes), as curiosity often drives the desire to learn more about any topic.

*Yes, I have heard about the Entrepreneurial Competence at my school. In some meetings we have received introductory information about key competences and how to identify opportunities in the competences. We have also talked about the topic of project planning.*

Primary School Teacher

*Entrepreneurial Competence has been an occasional topic at my educational centre. During some discussions, we have focused on basic ideas that also address other key competences, such as resource management and problem solving.*

University Teacher

*I have heard that the entrepreneurial competence refers to basic knowledge on how to explore new business models and assess their viability in a dynamic market. However, I believe that this competence is not only limited to the business domain; it is also very relevant in the educational context.*

Secondary School Teacher

Education professionals underlined the importance of enhancing certain key skills associated with the EC (14 quotes) for entrepreneurial success.

*Among the key skills that make up the entrepreneurial competence, I have heard about the ability to adapt and the cultivation of initiative to face entrepreneurial challenges.*

University Teacher

*When I think of entrepreneurial competence, I immediately think of skills such as problem solving, teamwork and critical thinking.*

Secondary School Teacher

Specifically, participants highlight that critical-thinking and problem-solving skills have always been essential for advancing in society. In general, they see these skills as a real opportunity to improve both in education and in their professional lives. Their opinions and attitudes (12 quotes) reflect a positive view of the importance of fostering the EC.

*I recognize that I have heard of the entrepreneurial competence, but I think that it is not given enough importance in educational centres. If we think about it, the entrepreneurial competence has become an essential aspect inside and outside the classroom. This competence provides students with valuable tools to face a constantly changing world.*

Research Staff

In terms of specific details (10 quotes), some participants shared their practical experiences of incorporating elements of the EC in their teaching.

*I consider it essential to transcend institutional barriers in education. My approach involves strengthening connections with the community through collaborations in community service projects. We actively participate in Service-Learning Projects, where students apply their entrepreneurial competences to address local problems.*

University Teacher

Finally, comparisons with other educational centres (five quotes) sparked interest in adopting similar approaches. Teachers expressed interest about how other educational centres implemented entrepreneurial initiatives and the potential benefits for their own students.

*Comparison with other educational centres has led us to implement specific entrepreneurial-skills-development workshops, which are strengthening students’ confidence to take the initiative in group work and apply their knowledge in real-world contexts.*

Primary School Teacher

### 3.2. Second Category of the Interview: Teaching of the Entrepreneurial Competence

The second category of the interview, consisting of eight codes and 110 quotes (see Table 3), focused on exploring how the EC is developed within educational contexts.

Based on the results obtained, it can be stated that there is a lack of communication (30 quotes) about how the EC is promoted in the educational centres, which leads to a lack of knowledge about its teaching (14 quotes). This communication problem stands out as one of the main barriers to the correct development of the EC in the classroom.

*At my school we observe a lack of clear communication on how to develop this competence. I think this generates some confusion among teachers, and it would be beneficial to improve communication to align educational strategies.*

Primary School Teacher

*It is very difficult to teach this competence, inside or outside the subjects, as there is a lack of communication and coordination that impacts on the planning and execution of activities related to this competence.*

Secondary School Teacher

*In my research I have identified a significant pattern in relation to the lack of communication in the development of entrepreneurial competence. I think there is a disconnect between management and education professionals in relation to strategies for implementing entrepreneurial education.*

Research Staff

*Teachers do not receive information about entrepreneurial competence and we do not hold meetings to share practices that address it in the classroom. I strongly believe that the lack of communication and insufficient information about the results of implementing this competence generates uncertainty.*

University Teacher

Another point highlighted is the lack of specific teaching (18 quotes), together with the need to improve its development (25 quotes). Participants agree that there are no clear criteria or adequate resources to develop the EC into the educational curriculum.

*Every teacher interprets teaching by competences differently. In my opinion, we need a clear framework and a series of specific resources to guarantee effective teaching of the key competences, including entrepreneurship.*

Primary School Teacher

*I have no specific strategies or resources to implement quality entrepreneurial education. I see then that there is a lack of clear approaches to teaching by competences.*

University Teacher

*We need to actively seek more effective strategies and up-to-date resources to achieve the different competences and thus reflect the desired impact in the classroom.*

Research Staff

*The teaching of Entrepreneurial Competence needs to be thoroughly reviewed. The first step in implementing it effectively in the teaching and learning process is recognizing the need for improvement in the way it is taught to students.*

University Teacher

On the other hand, some educational professionals point out that, although the EC is mentioned in educational planning, there are no specific strategies or resources for its development in the classroom. Faced with these deficiencies, some educational centres have opted to foster the EC in their own subjects in a cross-cutting manner (10 quotes). In addition, some educational centres have resorted to projects and activities (seven quotes) that allow students to strengthen the EC in an active way. To a lesser extent, the EC has been taught independently (three quotes), through tutoring or specific programmes outside conventional subjects.

*The competence is developed through the practical application of skills such as creative thinking, time management and communication in various subjects, in a cross-cutting manner.*

University Teacher

*Entrepreneurial competence is developed through practical activities that implement initiatives which develop initiative and proactivity, using the Learning by Doing method.*

Secondary School Teacher

*The competence is taught through innovation programmes of the Regional Ministry of Education, orienting it towards divergent thinking.*

Primary School Teacher

*The competence is carried out through guidance and tutoring, independently of the subjects.*

University Teacher

*Sometimes we do projects that I think could be related to the entrepreneurship competence, but it is not something official. I would like to see more specific activities.*

Primary School Teacher

### 3.3. Third Category of the Interview: Activities and Development of the Entrepreneurial Competence

The third category of the interview, consisting of five codes and 62 quotes (see Table 4), focused on investigating whether activities are carried out in educational centres to foster the EC and what types of activities are implemented.

Based on the results, participants highlighted that the most prominent teaching and learning tools (12 quotes), which contribute to the EC, include digital (20 quotes) and paper-based resources (16 quotes). However, they emphasize the need to give greater importance to the EC, viewing it as an area still pending attention, which should be explicitly addressed in the classroom.

*Although we live in a digital age, I’ve seen that my educational centre promotes entrepreneurial competence through activities and case studies, included in various books, journals, and manuals, which foster critical thinking, proactivity, teamwork, and imagination. The drive for entrepreneurial competence is implicit, but at least it’s there.*

University Teacher

*In my classes, I see how digital educational resources allow entrepreneurial concepts to be applied in innovative ways. When I talk about entrepreneurial concepts, I’m referring to terms like creativity, decision-making, and problem-solving. I think that entrepreneurial competence is somehow developed through online educational games, interactive websites, and various learning apps. Even so, more importance should be given to this area.*

Primary School Teacher

*At my high school, entrepreneurial competence is implicitly included through guides, books, and paper-based activities that include practical, real-life situations that stimulate problem-solving and creativity. These exercises, included in various educational resources, enable students to face real entrepreneurial challenges.*

Secondary School Teacher

*Today, digital resources are so accessible and versatile they make it easier for us to incorporate learning experiences in the classroom that allow us to develop different key competences. In this case, digital resources play a crucial role in developing entrepreneurial competence, as they stimulate students’ entrepreneurial thinking and their creativity.*

Primary School Teacher

*Through various research projects and my own classes, I have discovered that case studies and lesson plans are essential for students to strengthen their understanding and practical application of certain entrepreneurial concepts: innovation, creativity, decision-making, adaptability, leadership, ethics, etc.*

Research Staff

Additionally, the cross-pollination of teaching practices (10 quotes) as well as collaboration with other organizations (four quotes) has been shown to foster the creation of an entrepreneurial environment.

*During the exchange of teaching practices that takes place during the meetings held at our center, a result of its establishment as a learning community, a very valuable synergy is created between colleagues, which allows us to strengthen our methods for developing entrepreneurial competence in our students.*

Primary School Teacher

*Collaboration with other institutions is a key activity for the development of fundamental competences in the 21st century. This collaboration allows us to expand the educational approach to entrepreneurial competence developed at my centre and allows us to provide enriching and practical opportunities for students.*

Research Staff

## 4. Discussion and Conclusions

The results of this study highlight an increasing need to explicitly focus on the EC into the educational environment (Lackeus, 2015). Although the participants recognize the impact of the EC and have taken steps to implement it with the help of both digital and paper-based resources, this competency still needs to be developed more deeply and systematically in educational programmes.

In this sense, education professionals acknowledge the lack of specific teacher training and agree with the need to receive continuous training on EC. This correlates with research by Sobrado and Fernandez (2010), who highlight the importance of incorporating skills such as creativity, problem solving and initiative in the teaching process. Tools such as Scratch, Minecraft Education, Book Creator, Perusall and Nearpod are presented as key resources to foster these skills (Paredes-Chacin & Florez-Ortega, 2024; Ramirez-Montoya & Gonzalez-Padron, 2022; Vega, 2015).

Although pedagogical tools such as digital and paper-based resources are identified as essential for the development of the EC, the study participants point out that these must be complemented with methodologies that allow the application of entrepreneurship concepts in a practical and contextualized way. For these reasons, methodologies such as Project Based Learning (PBL) and the Learning by Doing approach have proven to be effective in getting students to face real situations that require the use of skills related to the EC, such as decision making and teamwork (Angus, 2020; Heinonen & Poikkijoki, 2006).

The main challenge lies in the absence of strategic planning to effectively integrate these tools and methodologies into a structured and coherent curriculum. In this context, the EC cannot be meaningfully acquired by merely focusing on individual skills or through the isolated teaching of theoretical concepts (Rodrigues, 2023). Addressing this challenge requires a renewal of pedagogical approaches, thoughtful curricular design, and increased attention to teacher training needs (Coll & Martin, 2021).

In the light of these results, teachers are increasingly interested in the EC because they recognize its importance in today’s education (Bacigalupo et al., 2016; Saranza et al., 2022). However, this growing interest is not sufficient to ensure its effective development. Theoretical knowledge about the EC needs to be transformed into pedagogical practice, which requires a change of mindset and support resources (Charron & Rivera-Cruz, 2019; Rodrigues, 2023). This involves linking content and context, building bridges between theory and practice, and creating opportunities for teachers and students to apply their knowledge in real contexts (Bell & Bell, 2020; Crespi et al., 2022).

It is evident that communication, in a pedagogical sense, cannot be limited to the transmission of information. Communication must include processes of exchange, interaction, and dialogue, as well as processes of joint elaboration of meanings (Liu et al., 2022; Nuñez & Nuñez, 2018). To this end, the lack of communication and collaboration between educators and other members of the educational community hinders the long-term impact of EC (European Commission, 2011; Lilischkis et al., 2021). This individualistic tendency limits the collective construction of knowledge necessary for effective Entrepreneurship Education. Thus, communication and exchange of practical knowledge among teachers are fundamental to improve key skills, such as planning and persuasion, essential elements for the formation of entrepreneurial citizens (Liu et al., 2022; Perez, 2009).

Despite this growing interest, the development of the EC often relies on isolated teacher initiatives, which limits its scope and long-term sustainability. For this reason, a coordinated strategy at institutional and systemic level is necessary, which promotes shared planning and promotes a continuous dialogue on Entrepreneurship Education (European Commission, 2011; Vogel, 2017).

These challenges point to several practical implications that are particularly relevant for education professionals and policy makers (see Table 5).

Education professionals should receive continuous training in Entrepreneurship Education, as well as effectively implement active methodologies that foster key skills such as creativity, initiative and problem solving (Sobrado & Fernandez, 2010). On the other hand, policy makers should integrate interdisciplinary approaches into the curriculum in a structured way. They should also establish institutional support mechanisms and allocate strategic resources to ensure the effective development of the EC (Bacigalupo et al., 2016; Ngek, 2020; Seikkula-Leino et al., 2021). The implementation of collaborative teacher networks and the encouragement of innovation in pedagogical practices can further contribute to the EC at all levels of education (Bacigalupo & Cachia, 2011; El Atmani et al., 2023). In a broader sense, the EC enhances the overall quality of education by encouraging a shift towards more dynamic teaching methods that align with the demands of today’s labour markets (Corral-Joniaux et al., 2020; Nieto-Borbor & Martinez-Suarez, 2021). By adopting innovative pedagogical practices and using digital tools, educational institutions can provide students with the necessary resources to thrive in entrepreneurial environments (Patiño & Rodriguez, 2023; Salas et al., 2024).

In conclusion, the effective promotion of the EC requires an educational framework that emphasizes practice, innovation, and collaboration (Campos et al., 2023; Hernandez et al., 2015). Teacher training must be enhanced, focusing on both content knowledge and mastery of methodologies that promote the practical application of skills linked to the EC (Granados et al., 2020).

Currently, many education professionals, especially in higher education, still lack adequate training to promote Entrepreneurship Education, as well as a clear understanding of the appropriate pedagogical tools to do so (Ripolles, 2011). Therefore, it is crucial to guide teachers on organizational, curricular and psycho–pedagogical criteria in relation to the development of the EC in the classroom (Sobrado & Fernandez, 2010).

## 5. Limitations and Future Lines

This study has some limitations. First, its cross-sectional design restricts the ability to draw causal conclusions, as it captures participants’ perceptions at a single point in time. Second, cultural and educational differences among participants may have influenced their perceptions of EC. National curricula, teacher education models and institutional priorities vary considerably between countries and even between institutions within countries. These differences may have led to variations in the way that the EC is understood and developed. However, the inclusion of participants from diverse national and institutional contexts was intended to enrich the study by incorporating a variety of cultural and educational perspectives, rather than making direct comparisons between countries and educational centres.

Future research should explore the longitudinal impact of the EC on educational innovation, particularly assessing how this competency influences long-term institutional transformation. Comparative studies across different educational contexts and cultural settings may also offer a broader perspective on the adaptability and effectiveness of this competency.

Additionally, further research could focus on measuring the direct impact of the EC on student learning outcomes, fostering a more comprehensive understanding of its role in innovative education. Future studies could also examine the institutional factors, such as policies, support mechanisms and pedagogical approaches, that facilitate or hinder the development of the EC. Finally, cross-cultural dimensions could be further explored to better understand the role of local educational frameworks in the fostering of the EC.

## Figures and Tables

**Table 1:** Distribution of questions, categories, codes and quotes for the first section of the study.

Questions	Categories	Codes	Quotes	%
Q1 = Are you familiar with the concept of EC? If yes, could you briefly describe what it means to you or what you know about it?	1. Knowledge and Perception of the EC	6	85	33.1%
Q2 = Is the EC developed as an independent subject at your educational centre? If yes, could you briefly describe how it is developed?	2. Teaching of the EC	8	110	42.8%
Q3 = Is the EC developed within other subjects at your educational centre? If yes, could you briefly describe how it is developed?				
Q4 = Are specific activities or practices carried out at your educational centre to develop the EC? If yes, could you briefly describe what kind of activities and practices are carried out?	3. Activities and Development of the EC	5	62	24.1%
TOTAL		19	257	100%

Abbreviations: EC, Entrepreneurial Competence. Source: own elaboration.

**Table 2:** Category “Knowledge and Perception of the Entrepreneurial Competence”.

Category	Codes	Quotes	%
Knowledge and Perception of the Entrepreneurial Competence	Interest and curiosity	25	29.4%
	Theoretical knowledge	19	22.4%
	Key skills	14	16.5%
	Opinions and attitudes	12	14.1%
	Specific details	10	11.8%
	Comparison with other educational centres	5	5.9%
	TOTAL	85	100%

Source: own elaboration.

**Table 3:** Category “Teaching of the Entrepreneurial Competence”.

Category	Codes	Quotes	%
Teaching of the Entrepreneurial Competence	Lack of communication	30	27.3%
	Need to improve its development	25	22.7%
	Lack of specific teaching	18	16.4%
	Lack of knowledge about its teaching	14	12.7%
	Subjects	10	9.1%
	Projects and activities	7	6.4%
	Independence teaching	3	2.7%
	Comparison with other centres	3	2.7%
	TOTAL	110	100%

Source: own elaboration.

**Table 4:** Category “Activities and Development of the Entrepreneurial Competence”.

Category	Codes	Quotes	%
Activities and Development of the Entrepreneurial Competence	Digital resources	20	32.3%
	Paper-based resources	16	25.8%
	Teaching and learning tools	12	19.4%
	Cross-pollination of teaching practices	10	16.1%
	Collaboration with other organisations	4	6.4%
	TOTAL	62	100%

Source: own elaboration.

**Table 5:** Practical implications for the development of Entrepreneurial Competence.

Stakeholders	Practical Implications
Education Professionals	Receive continuous training in Entrepreneurship Education.
	Promote collaboration and exchange of experiences among teachers.
	Apply a contextualized approach in the use of pedagogical tools.
	Effectively implement active and innovative methodologies.
Policy Makers	Integrate interdisciplinary approaches into the curriculum.
	Establish institutional support mechanisms.
	Allocate strategic resources.
	Promote pedagogical innovation at all levels of education.

Source: own elaboration.

## Data Availability

Due to the anonymity and confidentiality of the data obtained, the authors have not reported any of the data obtained, the purpose of which is exclusively the development of this research.
